# Improving Carers’ Engagement for Patients Admitted to Psychiatric Intensive Care Unit (PICU): A Quality Improvement Project

**DOI:** 10.1192/bjo.2024.413

**Published:** 2024-08-01

**Authors:** Simona Hossain, Lois Clark, Emily Pettifor, Shantala Satisha, Sua Youn

**Affiliations:** Willow Suite Psychiatric Intensive Care Unit, Littlebrook Hospital, Dartford, United Kingdom

## Abstract

**Aims:**

The project aims to improve carers’ engagement for patients admitted to our male Psychiatric Intensive Care Unit by improving communication between staff and carers; and by involving carers more in patients’ care.

Hypothesis:

Among patients admitted to PICU, there is inconsistency in communication with carers and in involving carers in patients’ care. We expect an improvement in these parameters with the quality improvement project.

Background:

Within PICUs, patients with severe psychiatric illness face social isolation. Challenges arise when carers are not engaged, impeding patient support and personalised care. Involving carers becomes crucial for informed decision-making, ensuring both patients and carers actively participate in the care process. National Association of PICUs and The Royal College of Psychiatrists' Guidance for PICU sets out recommendations regarding timelines and types of interventions to be offered to carers.

**Methods:**

Initial baseline data was collected by reviewing patient electronic notes.

We then tested interventions to improve carers’ engagement by using the Plan-Do-Study-Act (PDSA) methodology over 2 cycles. In the first cycle, we engaged the nursing staff by presenting the baseline data and recommendations to improve carers’ engagement. In the second cycle, we introduced an admission protocol to ensure carers were engaged consistently. The parameters assessed were documentation of carers details; contacting carers within 24 hours of admission; documenting carers' views in care plan; inviting carers to Care Plan Approach (CPA) meetings and offer an appointment for carers with staff.

Data was collected after each PDSA cycle to monitor change.

**Results:**

Of the patients admitted to PICU, 29% had their carers’ details documented at baseline, 40% after the first PDSA and 80% after the second PDSA. 42% of carers were contacted within 24 hours of admission at baseline; 66% and 30% after the two PDSA. 50% of carers had their views included in the care plan at baseline; 0% and 30% after the interventions. At baseline, 42% of patients’ carers were invited to the CPA meeting, 66% and 30% after the two PDSA cycles. 50% of patients’ carers were offered an appointment with staff at baseline, 66% and 30% after the two interventions.

**Conclusion:**

As a result of this quality improvement project there has been an improvement in engaging carers of patients admitted to PICU. This was not sustained for the second cycle due to many regular senior staff being on leave during Christmas. The next steps will be to implement this consistently and produce a carers’ information pack.

